# Health-related quality of life and socioeconomic status: inequalities among adults with a chronic disease

**DOI:** 10.1186/1477-7525-12-58

**Published:** 2014-04-25

**Authors:** Andreas Mielck, Martin Vogelmann, Reiner Leidl

**Affiliations:** 1 Helmholtz Zentrum Muenchen, German Research Center for Environmental Health, Institute of Health Economics and Health Care Management, Neuherberg D-85758, PO Box 1129, Germany; 2 Wort & Bild Verlag, Konradshöhe GmbH & Co. KG, Baierbrunn, Germany; 3 Munich Center of Health Sciences, Ludwig-Maximilians-University, Munich, Germany

**Keywords:** EQ-5D-3 L, Chronic disease, Socioeconomic status, Health inequalities, Germany

## Abstract

**Background:**

A number of studies have shown an association between health-related quality of life (HRQL) and socioeconomic status (SES). Indicators of SES usually serve as potential confounders; associations between SES and HRQL are rarely discussed in their own right. Also, few studies assess the association between HRQL and SES among those with a chronic disease. The study focuses on the question of whether people with the same state of health judge their HRQL differently according to their SES, and whether a bias could be introduced by ignoring these differences.

**Methods:**

The analyses were based on a representative sample of the adult population in Germany (n = 11,177). HRQL was assessed by the EQ-5D-3 L, i.e. the five domains (e.g. ‘moderate or severe problems’ concerning mobility) and the Visual Analog Scale (VAS). SES was primarily assessed by educational level; age, sex and family status were included as potential confounders. Six chronic diseases were selected, each having a prevalence of at least 1% (e.g. diabetes mellitus). Multivariate analyses were conducted by logistic and linear regression.

**Results:**

Among adults with a chronic disease, most ‘moderate or severe problems’ are reported more often in the low (compared with the high) educational group. The same social differences are seen for VAS values, also in subgroups characterized by ‘moderate or severe problems’. Gender-specific analyses show that for women the associations with VAS values can just be seen in the total sample. For men, however, they are also present in subgroups defined by ‘moderate or severe problems’ or by the presence of a chronic disease; some of these differences exceed 10 points on the VAS scale.

**Conclusions:**

Low SES groups seem to be faced with a double burden: first, increased levels of health impairments and, second, lower levels of valuated HRQL once health is impaired. These associations should be analysed and discussed in their own right, based on interdisciplinary co-operation. Social epidemiologists could include measures of HRQL in their studies more often, for example, and health economists could consider assessing whether recommendations based on HRQL scales might include a social bias.

## Background

Health-related quality of life (HRQL) is often assessed by the EuroQol 5D (using here the version with three problem levels, EQ-5D-3 L). The EQ-5D-3 L has been translated into many languages and applied to numerous health problems (more than 3,900 publications are currently listed on the homepage) [1]. Associations with socioeconomic status (SES) are reported in a number of them, but they are rarely discussed in their own right. Indicators of SES (e.g. educational level) usually serve as potential confounders, and some studies do not even mention the associations between SES and HRQL, although they are reported in the tables. Another reason for including SES is to demonstrate the construct validity of the instrument for assessing HRQL, but associations between SES and HRQL are an important topic per se. Concerning research focusing on health inequalities, it has to be asked whether these inequalities are underestimated if HRQL is not included in the analyses. Concerning research focusing on quality of life, it has to be asked whether people with the same state of health judge their HRQL differently according to their SES, and whether this could lead to a social bias introduced by assessing HRQL with an instrument such as the EQ-5D-3 L. However, these questions are rarely addressed [2].

Three groups of analyses can be differentiated: (a) studies looking at differences in HRQL by SES in a sample of the general population; (b) studies reporting these associations after controlling for risk factors and for chronic diseases; and (c) studies showing these associations among those with a chronic disease. Most papers belong to the first group, and they often use the EQ-5D-3 L to assess HRQL. They come, for example, from the USA [3-6], New Zealand [7], Japan [8], China [9], the UK [10-13], Sweden [14], Spain [15,16], Greece [17,18], Denmark [19,20] and the Netherlands [21]; some studies combine data from different Western European countries [22-24]. Of course they also differ in the way the sample was drawn, but despite these differences they consistently show that HRQL increases with increasing SES, in terms of problems reported [9,12,19-21,23,24] and in terms of overall HRQL assessment [3-6,10,13,14,16,17,22].

There are also a number of papers looking at the association between SES and HRQL after controlling for risk factors and chronic diseases (second group), and again most of them have assessed HRQL using the EQ-5D-3 L. They are based on survey data from the general population, controlling, for example, for risk factors such as obesity and smoking [25], for self-reported diabetes [26], for the presence of any chronic disease [2], for a selection of different chronic diseases [27-30] or for a number of chronic diseases [31-33]. The studies come from the USA and Canada [26,27,32,33], the UK [25,31], Sweden [28], Finland [29] and Germany [2,30]. And again, despite differing countries and samples, they consistently show that low SES is associated with low HRQL even after controlling for these risk factors and chronic diseases, in terms of both problems reported [2,28] and overall HRQL assessment [25-27,29-33].

Thus, the basic association between SES and HRQL seems to be well established and undisputed (i.e. HRQL is especially low in the low SES groups, in the total population and also in analyses adjusting for some risk factors or chronic diseases). The third group of analyses is more interesting, however. If the same association between SES and HRQL could also be found among patients with diabetes mellitus, for example, this would clearly indicate two potential problems: health inequalities would be underestimated if HRQL is not assessed, and policy recommendations based on HRQL analyses could be biased if the differences according to SES are not stressed. Although not primarily devoted to the investigation of SES, most of these studies include SES in the analysis of HRQL, looking at a specific group of patients such as patients with heart attack [34], myocardial infarction [35], type 2 diabetes [36], chronic low back pain [37], chronic obstructive pulmonary disease (COPD) [38], depression [39] or patients undergoing elective cardiac surgery [40] or percutaneous coronary intervention (PCI) [41]. Other studies are based on population surveys including different chronic diseases [33,42,43]. Again, HRQL is mostly assessed using the EQ-5D-3 L. The associations with SES are often reported in simple bivariate analyses, and the results are contradictory. Some report that the positive association between SES and HRQL is significant either for men or for women or for specific indicators of SES [37]. Others show that this association is significant only for the EQ-5D-3 L time trade-off (TTO) valuation, but not for the EQ-5D-3 L Visual Analog Scale (VAS) [40]. Some indicate that this association is not statistically significant [39], and some do not even include a statistical test for this association [33,42].

In this third group, the association between SES and HRQL does not become any clearer in analyses controlling for other variables such as age. Jerant et al. [44] analysed data from chronically ill adults enrolled in a self-management intervention, and they found no significant association between educational level and EQ-5D-3 L summary index. Lacey and Walters [34] analysed data from patients who had a heart attack, assessing HRQL using EQ-5D-3 L and SF-36. Concerning EQ-5D-3 L, they found that HRQL (summary index) is lower in the low SES group, but the 5% level of statistical significance was clearly missed. Concerning SF-36, a significant association with SES could just be found for two domains (i.e. bodily pain and vitality). Miravitlles et al. [38] analysed data from patients with COPD. The results show that low educational level is associated with low HRQL (summary index), but no association was found if SES was assessed by occupational status. Schweikert et al. [35] analysed data from patients with myocardial infarction (MI), and Wexler et al. [36] analysed data from patients with type 2 diabetes. They both found that HRQL (summary index) was significantly lower in the low SES group. Stafford et al. [43] published an analysis based on survey data, including a number of different chronic diseases. Using the EQ-5D-3 L (summary index), they found that the reduction in HRQL resulting from hypertension or diabetes was significantly greater in the low compared with the high SES group, and that this association could not be found for angina, heart attack or stroke. But again, in these analyses, SES is mostly included as a confounder, and the associations between SES and HRQL are not discussed in any detail.

We would argue that the association between SES and HRQL among patients with a chronic disease is an important topic in its own right, and to date little is known about it. It has rarely been addressed, and the available studies show mixed and sometimes contradictory results. In order to support this discussion, the objective of our study is to add a detailed analysis from Germany, testing the hypothesis that HRQL is not especially low in low SES groups, in the total population or in subgroups of the population defined by a chronic disease.

## Methods

The HRQL questions have been included in a yearly representative survey of the German adult population, conducted by the ‘Wort & Bild Verlag’ 2006–2011. The sample was drawn based on a random selection of households and from one person over 14 years of age per household. The overall response rate was about 73% [2]. In order to account for potential selection bias, a weighting factor based on the distribution of age and sex has been included in all analyses to ensure representativeness. Each survey comprises about 2,000 individuals aged 14 years or above. The analyses were restricted to participants who were at least 20 years of age, as school education (our main independent variable) should be finished by this age. The surveys comprise a face-to-face interview and a self-administered questionnaire for the EQ-5D-3 L. Previous publications based on other parts of these data describe the distribution of HRQL in Germany [45] and the basic associations with educational level [2]. The data have also been used to develop a new method estimating a value set for the EQ-5D-3 L based on health states experienced [46].

Concerning studies on health inequalities in Germany, educational level is widely accepted to be the most important indicator of SES, mainly for two reasons: Educational level is crucial for future occupation, and educational level rarely changes after 20 years of age. The German educational system is quite complicated and it differs between the 16 German states. The main characteristics are: (a) three levels of school (in German: Hauptschule, Mittlere Reife, Abitur) with the highest level (i.e. ‘Abitur’ with a standard duration of 12 or 13 years) qualifying for the university, and (b) three levels of vocational training: no vocational training, blue collar, white collar (e.g. with university degree). The distribution in the population strongly depends on the age group under study. The percentage of adults with the lowest level of school (i.e. ‘Hauptschule’) is steadily decreasing. Our study focuses on adults with a mean age of about 50 years. The statistical report for Germany states that in this age group the percentage with ‘Hauptschule’ is about 40%, whereas it is just about 20% in the age group 25–35 years [47]. In our study, educational level was assessed by a single question, primarily assessing the highest level of school education (i.e. adding hardly any information on vocational training). This is why we focused on school education, differentiating between ‘low’ (i.e. ‘Hauptschule’) and ‘high’ (i.e. ‘Mittlere Reife’ or ‘Abitur’). The percentage of participants with ‘Hauptschule’ is quite high, but the German school system does not allow for a narrower definition of low SES. It is difficult to fit the German educational system into the International Standard Classification of Education (ISCED). A substantial effort has been made, for example, in an international study concerning genetic variants associated with educational attainment [48]; finally ‘Hauptschule’ has been categorized as ISCED 2 or 3.

Additional analyses were carried out with monthly per-capita income instead of educational level, differentiating between five groups each including about 20% of the sample; finally, ‘low income’ (quintiles 1 and 2) was compared with ‘high income’ (quintiles 4 and 5). The dataset also includes three other sociodemographic variables, i.e. nationality, family status and employment status. Concerning nationality, more than 98% of the participants reported ‘German’. This is why nationality has not been controlled for in further analyses. Two groups of family status could be differentiated, i.e. living with a partner and living without a partner (68.42% resp. 31.58%). Employment status was assessed by six categories: (a) full-time or part-time employed (53.01%); (b) not employed due to military or civil service, maternity leave (0.87%); (c) temporary unemployed (4.46%); (d) pensioner (30.62%); (e) not employed due to housekeeping (e.g. housewife) (7.28%); (f) not employed due to vocational training, occupational re-training (3.76%). Some studies show an association between lack of paid employment and low HRQL, again most of them using the EQ-5D-3 L [23,49,50]. In our study, lack of paid employment was defined by combining the last four categories mentioned above (comprising 46.99% of the sample).

Concerning morbidity, the participants were asked whether they had a chronic disease, and we selected those six diseases with a prevalence of at least 1% (i.e. problems with the musculoskeletal system, hypertension, diabetes mellitus, heart disease, rheumatism, headache or migraine). In order to assess multi-morbidity, we included two more variables: first, ‘any of these six chronic diseases’ and, second, ‘any chronic disease’ (these six diseases plus other chronic diseases such as arthrosis, asthma, dermatosis or gastro-intestinal disease).

The dependent variables are provided by both parts of the EQ-5D-3 L, with the first part including five domains (i.e. mobility, self-care, usual activities, pain/discomfort, anxiety/depression) with three potential answers each (i.e. no, moderate or severe problems), and the second part using the Visual Analog Scale (VAS). Logistic regression is used for dichotomous dependent variables (i.e. the presence of moderate or severe problems), and linear regression for the continuous dependent variable (i.e. VAS), with other variables (e.g. age and sex) included as potential confounders. All analyses were conducted with the software package SAS ® version 9.1 (SAS Institute Inc., Cary, NC, USA).

## Results

The basic distribution of the variables is shown in Table 1. The dataset includes information from 11,177 people over 20 years of age with a slightly higher proportion of women (52.5%), reflecting their longer life expectancy. Many respondents belong to the low educational group (44%), a distribution always seen for adults in Germany [47]. Looking at the five dimensions of the EQ-5D-3 L, the prevalence of moderate or severe problems is highest for pain/discomfort and lowest for self-care. The prevalence of moderate or severe problems is higher for women than for men, though some of these differences are rather small. Concerning the six chronic diseases selected for further analyses, a significantly higher prevalence for women can just be seen for headache/migraine. The differences according to educational level are much clearer: moderate or severe problems are always considerably and significantly higher in the low compared with the high educational group, and the same picture is seen for just about all chronic disease variables.

**Table 1:** Prevalence of ‘moderate/severe problems’ and of chronic disease

	**Sex**				**Educational level**			
	**Men**		**Women**		**Low**		**High**	
	**n**	**%**	**n**	**%**	**n**	**%**	**n**	**%**
Total sample	5,305	47.5	5,872	52.5	4,927	44.0	6,250	56.0
Problems^a^ concerning:								
Mobility	709	13.4	958	16.3*	1,127	22.9	540	8.7*
Self-care	202	3.8	267	4.5	346	7.0	123	2.0*
Usual activities	567	10.7	756	12.9*	913	18.6	410	6.6*
Pain/discomfort	1,735	32.8	2,116	36.1*	2,264	46.0	1,586	25.4*
Anxiety/depression	449	8.5	930	15.9*	764	15.6	615	9.7*
At least 1 dimension	1,903	36.1	2,454	42.0*	2,477	50.7	1,881	30.3*
None of these^b^	3,363	63.9	3,387	58.0*	2,413	49.4	4,336	69.8*
Chronic disease^c^								
Muscul. system	381	7.2	426	7.3	487	9.9	320	5.1*
Hypertension	355	6.7	431	7.3	491	10.0	295	4.7*
Diabetes mellitus	198	3.7	236	4.0	291	5.9	142	2.3*
Heart disease	152	2.9	139	2.4	196	4.0	96	1.5*
Rheumatism	110	2.1	130	2.2	160	3.3	80	1.3*
Headache/migraine	59	1.1	169	2.9*	115	2.3	114	1.8
Any of these six^d^	972	18.3	1,156	19.7	1,283	26.1	844	13.5*
Any chronic disease^e^	1,298	24.5	1,675	28.5*	1,699	34.5	1,275	20.4*

*p < 0.05 (chi^2^ test comparing men/women resp. low/high educational level).

^a)^Moderate or severe problems; missing values: mobility (n = 17), self-care (n = 24), usual activities (n = 19), pain/discomfort (n = 22), anxiety/depression (n = 29).

^b)^None of these problems.

^c)^Selection of chronic diseases with an overall prevalence of n ≥2% (ordered by prevalence).

^d)^At least one of the six chronic diseases listed above.

^e)^Any chronic disease (these six plus other chronic diseases such as arthrosis, asthma, dermatosis or gastro-intestinal disease).

The distribution of the VAS values is shown in Table 2. The overall value is 80.0; it decreases considerably if moderate or severe problems concerning mobility etc. are reported or chronic diseases. Looking at the total sample, the value for men is higher than that for women, and the same differences can be seen in most subgroups defined by moderate or severe problems in at least one of the five domains. No significant differences between men and women could be found in the subgroups defined by chronic disease. The differences according to educational level are clear and consistent: low educational level is always associated with lower VAS values (in the total sample and in all subgroups).

**Table 2:** Mean VAS values in subgroups of participants with ‘moderate/severe problems’ or with chronic disease

		**VAS value (mean)**				
	**N**	**Overall**	**Sex**		**Educational level**	
			**Men**	**Women**	**Low**	**High**
Total sample	11,177	80.0	81.1	79.0*	75.3	83.7*
Problems^a^ concerning:						
Mobility	1,667	56.2	57.1	55.6	54.5	59.7*
Self-care	468	50.0	53.6	47.2*	47.2	57.7*
Usual activities	1,323	53.6	55.3	52.4*	52.3	56.7*
Pain/discomfort	3,850	64.9	65.6	64.3*	62.7	68.1*
Anxiety/depression	1,379	61.3	59.5	62.2*	57.6	65.9*
At least 1 dimension	4,358	66.3	66.6	66.0	63.7	69.7*
None of these	6,749	88.9	89.4	88.4*	87.3	89.8*
Chronic disease^a^						
Muscul. system	807	63.9	65.0	62.8	62.2	66.3*
Hypertension	786	63.2	63.7	62.8	60.9	67.0*
Diabetes mellitus	433	58.8	58.6	59.0	55.6	65.6*
Heart disease	292	57.0	56.7	57.4	54.8	61.6*
Rheumatism	240	59.3	60.8	58.0	57.0	64.0*
Headache/migraine	228	74.2	73.6	74.5	69.2	79.3*
Any of these six	2,128	64.5	64.5	64.5	61.8	68.6*
Any chronic disease	2,973	65.2	65.4	65.1	62.4	69.0*

*p < 0.05 (t-test comparing men/women resp. low/high educational level).

^a)^see Table 1.

Additional analyses (not shown in the table) looked at the associations from a different perspective, i.e. at the prevalence of chronic diseases by educational level in the subgroup not reporting any problems at all, a health state denoted ‘11111’ in the EQ-5D-3 L terminology. In this subgroup, the prevalence of ‘any of the six chronic diseases’ investigated in detail (see Table 1) is significantly higher in the low compared with the high educational group (8.13% versus 4.50%; p < 0.001). The same picture can also be seen for the prevalence of ‘any chronic disease’ (11.59% versus 7.78%; p < 0.001). Owing to reduced sample size by health states, this type of analysis was possible only for the subgroup denoted ‘11111’, but the results show that, among respondents not reporting any problem at all, the prevalence of chronic disease increases with SES.

The first group of multivariate analyses is based on the dependent variables ‘moderate or severe problems in the five domains of the EQ-5D-3 L’. Six multivariate analyses referring to the five problem domains of the EQ-5D-3 L or to the occurrence of at least one of them have been conducted for every group of participants (total sample, plus eight groups defined by ‘chronic disease’), and each model is adjusted for age and sex. The odds ratios for age and sex (not shown in the table) illustrate that most of these problems increase significantly with age (the only exception being anxiety/depression) and that most of these problems are significantly more common among women than among men (the only exception being pain/discomfort). The odds ratios for low compared with high educational level are shown in Table 3. Looking first at the total sample, moderate or severe problems concerning mobility are significantly higher for those with a low educational level, and the same is true concerning the other four domains. In the next step, the same association is tested for subgroups defined by the presence of a chronic disease. Among those with diabetes mellitus, for example, moderate or severe problems concerning mobility, self-care, usual activities and anxiety/depression are significantly more common for those with a low educational level. Similar differences according to educational level are seen for all chronic disease groups except rheumatism. Analysing these associations separately for men and women, most results are replicated, but some differences in health impact linked to educational level can be highlighted (not shown in the table). Relative to women, men with a low educational level have substantially higher odds of incurring ‘moderate to severe problems’ concerning self-care in five disease groups (musculoskeletal system, diabetes mellitus, heart disease, any of the six chronic diseases, any chronic disease). In contrast, women with a low educational level have substantially higher odds of incurring ‘moderate to severe problems’ concerning mobility, usual activities and pain/discomfort in the group with headache or migraine.

**Table 3:** Multivariate association between educational level and ‘moderate/severe problems’: participants with a chronic disease

	**Odds ratio (p-value) for low educational level **^**a**^					
	**Moderate or severe problems concerning …**					
	**Mobility**	**Self-care**	**Usual activities**	**Pain/discomf.**	**Anxiety/depression**	**At least 1 dimension**
Total sample	1.56*	1.87*	1.72*	1.45*	1.22*	1.39*
	(<0.0001)	(<0.0001)	(<0.0001)	(<0.0001)	(0.0012)	(<0.0001)
Chron. disease^b^						
Muscul.	1.30	2.11*	1.63*	0.95	1.10	0.81
System	(0.0998)	(0.0072)	(0.0038)	(0.8340)	(0.5968)	(0.4364)
Hypertension	1.02	1.49	1.53*	1.26	0.97	1.37
	(0.8887)	(0.1642)	(0.0170)	(0.1773)	(0.8862)	(0.0798)
Diabetes	1.58*	2.15*	2.26*	1.00	2.24*	1.47
Mellitus	(0.0484)	(0.0329)	(0.0007)	(0.9994)	(0.0018)	(0.1730)
Heart disease	1.93*	3.18*	2.05*	1.83*	1.48	1.77
	(0.0181)	(0.0031)	(0.0077)	(0.0464)	(0.1707)	(0.0824)
Rheumatism	1.72	1.72	1.55	2.15	1.21	2.09
	(0.0692)	(0.2001)	(0.1530)	(0.0843)	(0.5512)	(0.1963)
Headache/	1.91	2.28	3.04*	2.04*	1.73	1.79
Migraine	(0.0712)	(0.1777)	(0.0101)	(0.0488)	(0.1403)	(0.1157)
Any of	1.39*	2.36*	1.72*	1.32*	1.20	1.31*
These six	(0.0012)	(<0.0001)	(<0.0001)	(0.0132)	(0.0957)	(0.0235)
Any chronic	1.49*	2.07*	1.61*	1.31*	1.25*	1.28*
Disease	(<0.0001)	(<0.0001)	(<0.0001)	(0.0028)	(0.0145)	(0.0120)

*Significant at the 5% level.

^a)^Comparison group ‘high educational level’, adjusted for age and sex.

^b)^See Table 1.

In further analyses (not shown in table), educational level was replaced by per-capita income. The results are very similar as compared to those shown in Table 3, but the associations are somewhat weaker; just nine significant odds ratios (5% level) can be found in the six disease groups (as compared to 13 significant odds ratios seen in Table 3). A similar picture is seen if educational level is replaced by employment status. Concerning problems with ‘usual activities’, for example, lack of paid employment is associated with significant odds ratios in four disease groups (problems with musculoskeletal system: 3.27; diabetes mellitus: 3.50; heart disease: 6.02; headache/migraine: 6.09). In other analyses we included family status and employment status as independent variables. In the initial model with educational level as the dependent variable (see Table 3), most significant odds ratios remained significant at the 5% level (for example all significant odds ratios concerning problems with ‘usual activities’). Also, including family status and employment status hardly changed the odds ratios for per-capita income, and including family status hardly changed the odds ratios for lack of paid employment.

The second group of multivariate analyses considers the overall valuation of health. Table 4 presents the association between educational level and VAS values in different subgroups. Three multivariate analyses (men, women, both combined) have been conducted for every group of participants (total sample, plus seven groups defined by ‘problems’ and eight groups defined by ‘chronic disease’) and each model is adjusted for age. The VAS values are significantly lower in the low (compared with the high) educational group, for both men and women, but the difference is smaller for women (−1.84) than for men (−2.98). In the combined sample, this difference is also found for the subgroups defined by problem dimension (the only exception being usual activities) and by disease (the only clear exception being ‘musculoskeletal system’). For men, these associations can also be seen for the same subgroups defined by problem dimension and by disease. Some of these differences are pronounced, reaching more than 10 points on the VAS scale (i.e. moderate or severe problems concerning self-care; presence of diabetes mellitus, heart disease or headache/migraine). Among women, however, no significant differences could be found between low and high educational level in any subgroup.

**Table 4:** Multivariate association between educational level and mean VAS score: participants with ‘moderate/severe problems’ or with chronic disease

	**Linear regression coeff. for low educational level **^**a**^					
	**Men**		**women**		**Total **^**b**^	
	**Estimate**	**P-value**	**Estimate**	**P-value**	**Estimate**	**P-value**
Total	– 2.98*	<0.0001	−1.84*	<0.0001	−2.57*	<0.0001
Problems^c^ concerning:						
Mobility	– 4.11*	0.0064	−1.07	0.4683	−2.52*	0.0171
Self-care	−10.29*	0.0042	−2.25	0.4537	−5.69*	0.0134
Usual activities	– 3.06	0.0896	−0.60	0.7143	−1.72	0.1548
Pain/discomfort	– 2.86*	0.0020	−0.61	0.4821	−1.77*	0.0052
Anxiety/depression	– 5.00*	0.0116	−1.90	0.1643	−3.00*	0.0076
At least 1 dimension	– 2.92*	0.0007	−1.02	0.1920	−1.97*	0.0007
None of these	– 0.73*	0.0404	−0.43	0.2293	−0.58*	0.0218
Chronic disease^c^						
Muscul. system	– 0.82	0.6778	+0.26	0.8938	−0.24	0.8610
Hypertension	– 4.81*	0.0231	−0.65	0.7406	−2.74	0.0563
Diabetes mellitus	−10.14*	0.0006	−5.14	0.0533	−7.37*	0.0002
Heart disease	−10.53*	0.0092	−1.70	0.6290	−5.53*	0.0429
Rheumatism	– 7.18*	0.0345	−2.93	0.4447	−4.96	0.0532
Headache/migraine	−10.23*	0.0012	−3.73	0.1933	−5.54*	0.0151
Any of these six	– 4.74*	0.0002	−1.44	0.2197	−3.10*	0.0003
Any chronic disease	– 3.45*	0.0019	−0.86	0.3790	−2.15*	0.0034

*Significant at the 5% level.

^a)^Comparison group: ‘high educational level’; adjusted for age.

^b)^Also adjusted for sex.

^c)^See Table 1.

In further analyses (not shown in table), educational level was replaced by per-capita income. Low income is associated with lower VAS values, in the total sample, both among men and women. In the subgroups defined by ‘problems’ and by ‘chronic disease’, though, the sex specific results are quite different from those presented in Table 4: For educational level, the associations are mainly restricted to men (Table 4), for income, though, they are mainly restricted to women. The pattern does not change that much if educational level is replaced by employment status, i.e. lack of paid employment is associated with lower VAS values, and in the subgroups defined by ‘problems’ and by ‘chronic disease’ this differences is mainly found among men. In other analyses we included family status and employment status as independent variables. In the initial model with educational level as the dependent variable most significant odds ratios (see Table 4) remained significant. Also, including family status and employment status hardly changed the odds ratios for per-capita income, and including family status hardly changed the odds ratios for lack of paid employment.

## Discussion

The main results can be summarized in the following way: (a) Most ‘moderate or severe problems’ are reported more often in the low (compared with the high) educational group. This association can also be seen in multivariate analyses (e.g. controlling for age and sex) among adults with a chronic disease such as diabetes mellitus. (b) The VAS values are significantly lower in the low (compared with the high) educational group and also in subgroups defined by ‘moderate or severe problems’ or by the presence of a chronic disease. For men, the same picture can be seen in multivariate analyses (controlling for age), and some of these differences exceed 10 points on the VAS scale. For women, these associations were found only overall, but not at subgroup level. Given these results, the study hypothesis ‘no differences in HRQL according to SES in subgroups of the population defined by a chronic disease’ must clearly be rejected.

Some limitations need to be considered. The study is restricted to adults who are able to read and understand German. All information is based on self-report, including the information on chronic disease. It would be better to have subjective and objective measures of morbidity, but self-reported information does not have to be a major problem. It has repeatedly been shown, for example, that self-assessed health is a good predictor even for mortality [51,52]. Multiple testing could be an issue. Table 3, for example, comprises results from 54 different multivariate analyses. Following the Bonferroni correction, the p-value would have to be divided by the number of tests conducted (i.e. a p-value of about .001 would have to be used instead of .05). Just few p-values are smaller than .001, but such correction might be too conservative, as we would assume that the six models per disease group are not independent from each other (problems concerning ‘mobility’ could be associated with problems concerning ‘self care’, for example). Therefore, we do not report respectively corrected results, but we acknowledge that results will need confirmation in future research. The importance of the paper’s contribution lies in detailing educational impact on quality of life over a broad spectrum of morbidity; rather than elaborating on a specific chronic disease or on a specific ‘problem’ such as mobility. Educational impact is analysed across several groups of adults with different chronic diseases and health problems.

It has already been stressed above that there are few studies analysing the association between SES and HRQL for adults with a specific chronic disease, and that they show mixed results (see Introduction). Concerning multivariate analyses controlling for age and sex, we were able to identify just three studies reporting significant associations between SES and HRQL. Focusing on myocardial infarction survivors in Germany, Schweikert et al. [35] found that low educational level is associated with low HRQL (as assessed by the EQ-5D-3 L summary score). The same association was reported by Wexler et al. [36] in their study on patients with type 2 diabetes in the USA, assessing HRQL using the Health Utilities Index-III. Stafford et al. [43] analysed data from the Health Survey for England and showed that the reduction in HRQL (as assessed by the EQ-5D-3 L summary score) resulting from obesity, hypertension or diabetes was significantly greater in the group with low (compared with high) occupational status. We believe that our study extends this scarce evidence in several important ways. First, this study is not based on a clinical population, but on a large representative population survey. Second, different subgroups of ‘moderate/severe problems’ and of chronic diseases were analysed and compared based on the same methods of sampling and disease identification. Third and most importantly, this paper addresses the question of whether differences in HRQL according to SES can also be found among patients with six specific chronic diseases. In most of the studies mentioned above, SES is included as a confounder, and differences by SES are a ‘by-product’ not discussed in any detail.

## Conclusions

The study indicates that HRQL and SES may be significantly associated, and that health inequalities are misrepresented if HRQL is not taken into account. Low SES groups seem to be faced with a double burden: first, increased levels of health impairments and, second, lower levels of valuated HRQL once health is impaired. We would conclude that all studies of HRQL in patients with chronic disease should consider measures of SES, that associations between HRQL and SES should be analysed separately for men and women, and that these associations should be discussed in their own right and be considered in interpretation. Questions for future research include the development of approaches to explain why patients with low SES valuate their HRQL to be worse than patients with high SES, given the same chronic disease. To improve public health, it is important to develop and evaluate approaches aimed at reducing the impact of SES on HRQL, such as preventive or health-promoting measures targeted especially at groups with low SES.

Interdisciplinary co-operation may support work on the above research questions and the understanding of the relation between HRQL and SES. To give just one example, social epidemiology provides expertise concerning the analysis of interactions between SES and health, and health economics brings in expertise in valuing health and in measuring the impact of interventions [53]. The relation between SES and HRQL may also play a role in health care decision making. Following Dolan et al. [54], most people agree that potential gains in HRQL could be ‘sacrificed’ for potential reductions in inequalities. Trade-offs between efficiency and equity linked to specific health care interventions remain to be quantified in future studies. For groups of chronically ill patients, this study shows that SES and HRQL are associated, and thus informs decision makers that the health impacts of interventions for the chronically ill may incur equity aspects.

## Abbreviations

COPD: Chronic obstructive pulmonary disease; EQ-5D-3 L: EuroQol 5D (version with 3 problem levels); HRQL: Health-related quality of life; PCI: Percutaneous coronary intervention; SES: Socioeconomic status; SF-36: Short form-36; TTO: Time trade-off; VAS: Visual analog scale.

## Competing interests

The authors declare that they have no competing interests.

## Authors’ contributions

AM, conceived the study and was responsible for drafting the manuscript, data analysis and interpretation of the data. MV, contributed to the study design and to the interpretation of the data. RL, participated in the design and co-ordination of the study and contributed to the interpretation of the data and to drafting the manuscript. All authors have critically read the draft of the paper and contributed to its intellectual content, and all have read and approved the final manuscript.
